# Ventricular entry during surgical resection is associated with intracranial leptomeningeal dissemination in glioblastoma patients

**DOI:** 10.1007/s11060-022-04166-6

**Published:** 2022-10-23

**Authors:** Francesca Battista, Giovanni Muscas, Francesca Dinoi, Davide Gadda, Alessandro Della Puppa

**Affiliations:** 1 Department of Neurosurgery, Department of Neuroscience, Psychology, Drug Area and Child Health (NEUROFARBA), Careggi University Hospital, University of Florence, Largo Palagi 1, 50137 Florence, Italy; 2 Department of Neuro-Radiology, Careggi Hospital and University of Florence, Florence, Italy

**Keywords:** Glioblastoma, Leptomeningeal dissemination, Ventricular entry

## Abstract

**Purpose:**

Glioblastoma (GBM) is associated with a poorer prognosis when leptomeningeal dissemination (LMD) occurs. Recently, the role of both ventricular entry (VE) during surgery and subventricular zone localization of tumors in promoting LMD in GBM patients has been debated. This article investigates the role of VE in causing LMD in GBM patients.

**Methods:**

We conducted a retrospective analysis of GBMs operated on at our Institution between March 2018 and December 2020. We collected pre- and post-surgical images, anamnestic information, and surgical reports.

**Results:**

Two hundred cases were collected. The GBM localization was periventricular in 69.5% of cases, and there was a VE during the surgical procedure in 51% of cases. The risk of post-surgical LMD in the case of VE was 16%. The rate of LMD was higher in the case of VE than not-VE (27.4% vs. 4%, p < 0.0001). The rate of LMD in periventricular GBM was 19% (p = 0.1131).

**Conclusion:**

According to our data, VE is an independent factor associated with a higher rate of post-surgical LMD, and the periventricular localization is not independently correlated to this negative outcome. Neurosurgeons should avoid VE when possible. The correct surgical strategy should be founded on balancing the need for maximal EOR and the risks associated with VE.

## Introduction

Glioblastoma (GBM) is the most frequent primary brain tumor in adults [1–4]. The current standard treatment, the Stupp protocol, employs post-surgical radiotherapy plus adjuvant chemotherapy and has improved median survival up to 16.7 months [5–8]. Many factors influencing the prognosis have been cleared, such as the tumor size, the spread through the corpus callosum, multifocality, and the extent of resection (EOR) [9–13]. Earlier studies have shown a shorter survival after diagnosis of leptomeningeal dissemination (LMD) (12–20 weeks [14]), with OS sinking to 6 months [5, 15]. The ventricular entry (VE) during surgical exeresis has a debated role in influencing the prognosis of GBM [16–20].

Neurosurgeons have speculated whether VE during GBM excision could favor the cerebrospinal fluid (CSF) dissemination of tumor cells [18, 19, 21, 22]. Due to the lack of clear scientific evidence, the safest surgical strategy has often been adopted, sometimes compromising the EOR. Subsequently, to the increasingly scientific solid demonstration of EOR as the main positive prognostic factor for GBMs and how greater EOR was associated with better outcomes [11, 23, 24], the problem of VE has been tackled again in the literature. At first, VE was associated with a higher rate of LMD and worst prognoses [16–19, 25]. Recent works [20, 26] aimed at distinguishing VE from primary subventricular zone (SVZ) localization of GBM, identifying the latter as the only factor linked to higher rates of LMD of GBM. The SVZ, a pluripotent stem cell niche in adults, is localized in the wall of lateral ventricles [20, 27, 28]. In the case of GBM invasion, it would be linked with disease progression [29]. Despite the latest reports supporting this hypothesis, the level of evidence is low [20].

Although the EOR should be as maximal as possible [11, 30–32], the effect of VE on GBM progression has to be clarified. Our work aimed to compare the post-surgery LMD associated with VE and SVZ localization to determine the risk associated with both factors and define the best surgical strategy in supratentorial GBM.

## Materials and methods

The prospectively collected electronic database of our Institute was retrospectively searched for surgically treated GBM (WHO grade IV) between March 2018 and December 2020. In all cases, the histological diagnosis was GBM without any other component (as PNET). Pre- and postoperative radiological exams (brain computed tomography [CT] and magnetic resonance imaging [MRI]), surgical and clinical reports, and histological diagnoses were retrieved. Brain imaging was performed on either a 3 T (Ingenia 3 T, Philips Medical Systems, Best, The Netherlands) or a 1.5 T (Magnetom Aera, Siemens Healthcare) MRI scanner. The protocol included bi-dimensional non-contrast and contrast-enhanced T1-weighted spin-echo (SE), T2-weighted SE, T2* gradient-echo (GE), and diffusion-weighted (DWI) sequences, plus three-dimensional (3D) Fluid Attenuated Inversion Recovery (FLAIR), non-contrast and contrast-enhanced T1 Turbo Field Echo (TFE) or Magnetization Prepared Rapid Gradient Echo (MPRAGE) sequences. Both 3D-FLAIR and T1-weighted imaging were obtained with 1 mm slice thicknesses. Postcontrast T1-weighted imaging was performed after the Dynamic Susceptibility Contrast Perfusion Weighted (DSC-PWI) sequence for perfusion imaging, acquired during intravenous administration of 0.1 mmol/Kg bolus of a gadolinium-based macrocyclic contrast agent (Gadoteridol).

**Fig. 1:**
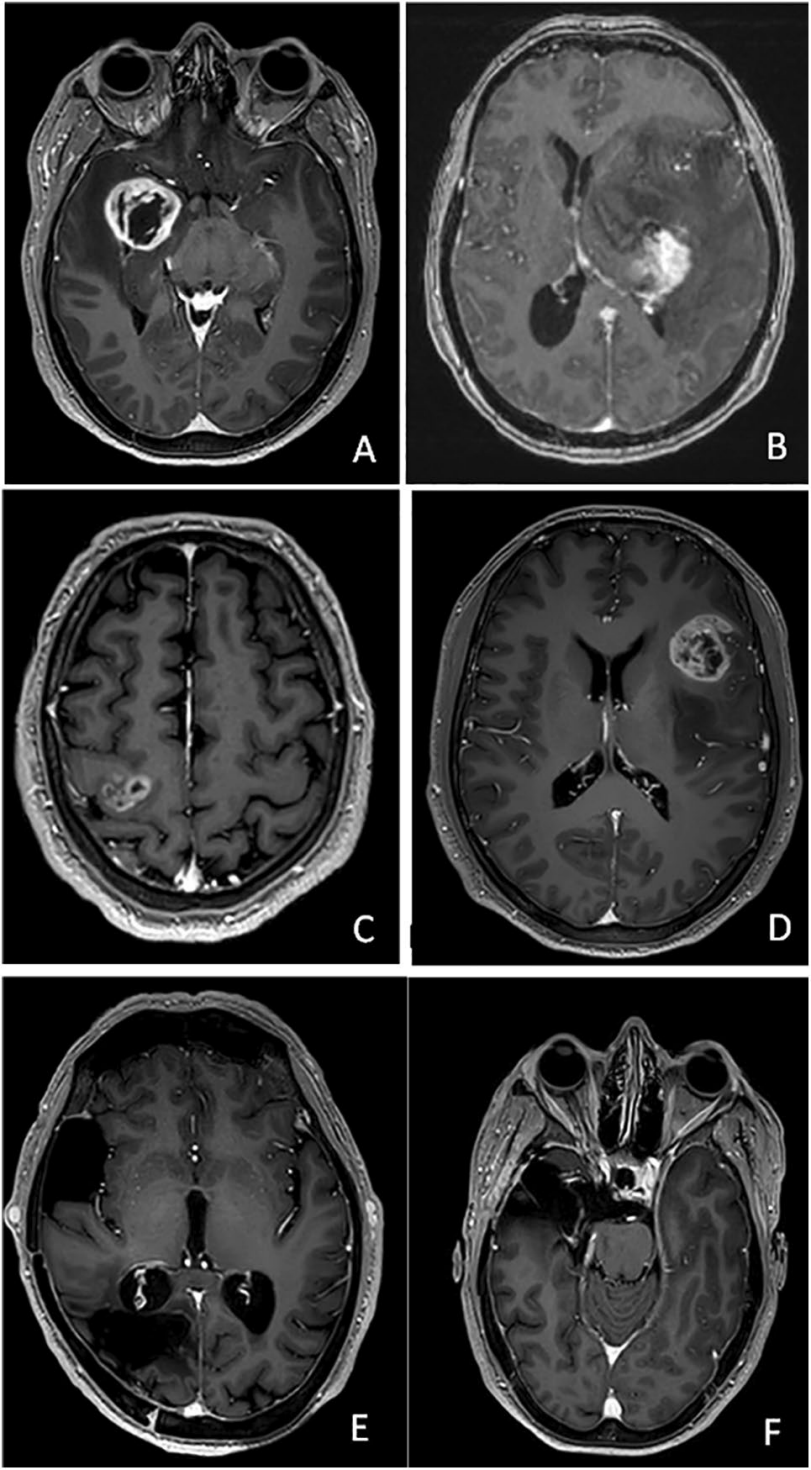
periventricular GBM (**A**, **B**); not periventricular GBM (**C**, **D**); VE (**E**, **F**); *VE* ventricular entry, *GBM* glioblastoma multiforme

Preoperative MRIs were examined in T1-weighted and gadolinium-enhanced T1-weighted sequences for identifying GBM localization (Fig. 1).

The SVZ localization was considered a contrast-enhanced area less than 1 cm from the ventricular wall without an apparent subependymal spread (Fig. 1A, B; C, D). Postoperative MRIs (at least one-month post-operatively to avoid confounding surgery-related alterations) were used to detect VE, which was considered present if a clear breach between ventricles and surgical cavities was observed(Fig. 1E, F). Surgical reports were screened if radiological images were inconclusive.

Postoperative brain MRIs (one month and six months after surgery) were examined to identify LMD, and FLAIR and gadolinium-enhanced T1-weighted sequences were analyzed by two independent observers (DG and FB), blinded to the violation of ventricular walls during surgery. We used a numerical code instead of the patients' names to identify them: this allowed us to examine the two scans independently, preventing a potential bias related to the previous knowledge of VE. The LMD was defined as leptomeningeal contrast enhancement along the contours of the gyri and sulci, as nodular enhancement in the subarachnoid space, or along the subependymal zone [15]. A six-month follow-up was considered the cut-off for the absence of LMD. Patients with consolidated disease recurrence at early postoperative imaging were excluded from the analysis since LMD could develop directly from the relapsing tumor.

To highlight the specific risk of LMD associated with VE, we analyzed the LMD rate in the subgroup of periventricular GBM. We subdivided this subgroup into VE and not-VE and calculated each LMD rate.

A forward and backward logistic regression analysis was performed to evaluate the association of periventricular location and VE with LMD, considering demographic data and, when available, molecular biomarkers (Isocitrate dehydrogenase [IDH]1/2 and ATRX gene mutations, O-6-Methylguanine-DNA Methyltransferase [MGMT] promoter methylation) as covariates. Variables with *p* values < 0.2 in the univariate analysis were included in the multivariate analysis. Odds ratios (OR) and corresponding 95% confidence intervals (95% CI) were thus estimated by a logistic regression model. Statistical analysis was performed with MedCalc (version 9.6.2.0; Mariakerke, Belgium). The statistical significance threshold was set at *p* < 0.05.

## Results

All results are summarized in Table 1.

**Table 1:** Final population characteristics (*GBM* glioblastoma multiforme, *VE* ventricular entry)

Total number	200
Sex	
- Male	110 (55%)
- Female	90 (45%)
Type of resection	
- First exeresis	191 (95.5%)
- Exeresis of recurrences	9 (4.5%)
Mean age	63.28 (± 10.32)
Mean time of follow-up (months)	8.6 (± 6.7)
Relationship with ventricle	
- Periventricular GBM	139 (69.5%)
- Periventricular GBM	61 (30.5%)
Ventricular entry	102 (51%)
Genotype	
- IDH mutant	11 (5.5%)
- Wild-type	39 (19.5%)
- Reported	150 (75%)
- Methylated	119 (59.5%)
- Non MGMT-methylated	69 (34.5%)
- Reported	12 (6%)
VE	
- In periventricular GBM	80 (58%)
- In not periventricular GBM	22 (36%)
LMD	
- VE	28 (27.4%)
- Not-VE	4 (4%)

During the study period, 200 patients (110 male and 90 female, 55% vs. 45%) with a median age of 63.28 years (± 10.32, range 21–86) underwent exeresis of intracranial GBM (191 patients [95.5%] were GBM of the first diagnosis; nine patients [4.5%] were recurrences).

The mean follow-up was 8.6 months (± 6.7). GBM localization was periventricular in 139 patients (69.5%) and far from the ventricle in 61 patients (30.5%). The VE was observed in 102 cases (51%), and MRI was inconclusive for VE in 10 of these patients. In these cases, we searched for VE in surgical reports, which was present in 6 of these 10 cases.

**Fig. 2:**
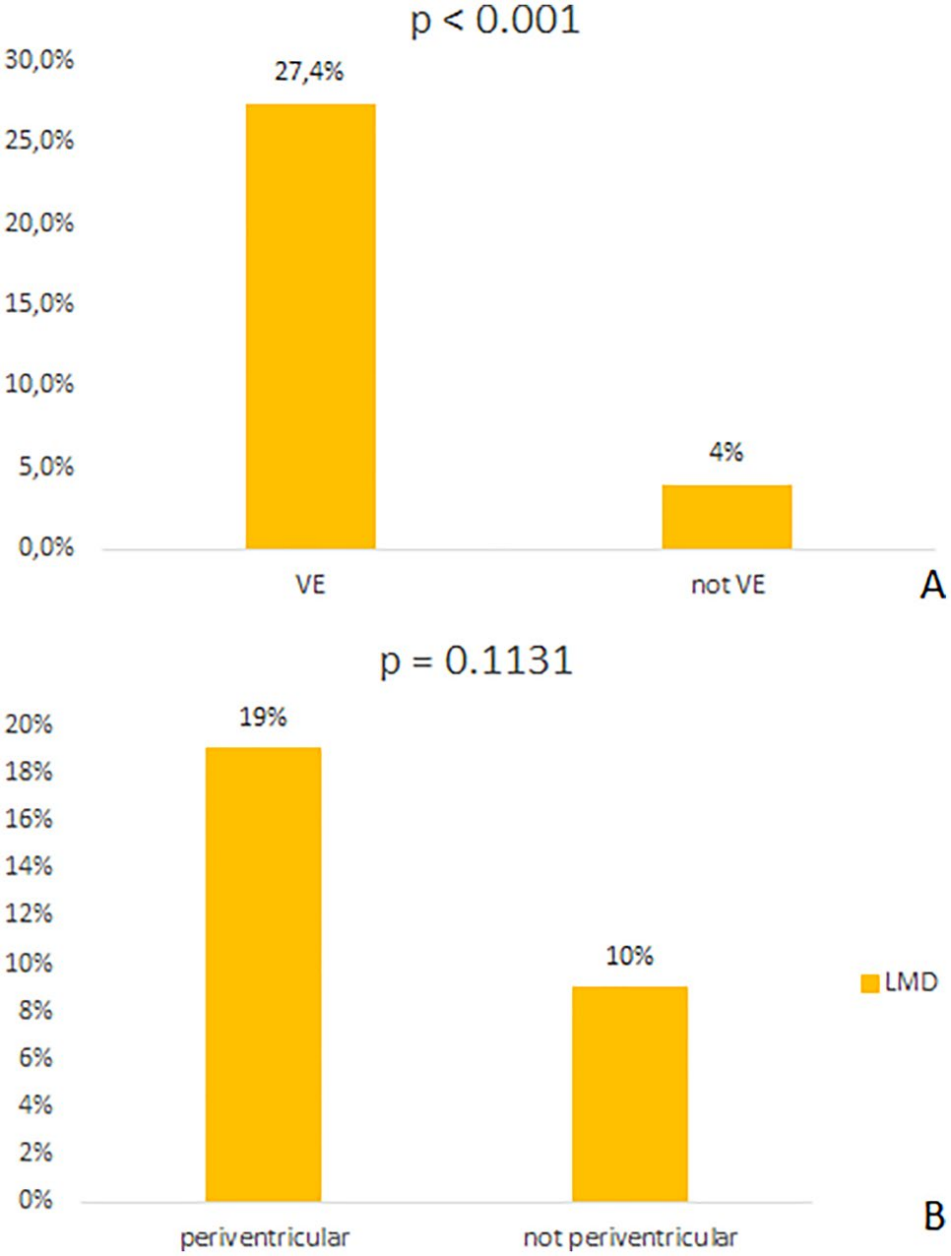
LMD in VE and not VE group (**A**). LMD in the periventricular and not the periventricular group (**B**). *LMD* leptomeningeal dissemination, *VE* ventricular entry, *not VE* not ventricular entry

The IDH status was available for 50 patients (25%), and an IDH-1 or -2 mutation was detected in 11 cases (5.5%). The MGMT promotor methylation was present in 119 cases (59.5%) and not reported in 12 cases (6%). The localization was periventricular in 139 cases (69.5%), and the VE was more frequent in peri-ventricular GBM (80 patients) than in cases of GBM far from the ventricle (22 patients) (58% vs. 36%). LMD was observed in 32 cases (16%): in 13 cases among these (40.6%), LMD arose between one and six months of follow-up, and in 19 cases (59.4%) occurred 6 months after surgery. LMD was strongly associated with VE (p < 0.0001) (see also Table 1). We did not find higher rates of post-surgical LMD in periventricular GBM: in this group, the rate of LMD was 19% (26 patients), and in non-periventricular GBM, it was 10% (6 cases) (p = 0.11) (Fig. 2).

Concerning VE during surgical exeresis, LMD rates in periventricular GBM was 31% when VE was performed (25 cases), whereas it was 4% when VE did not occur (1 patient) (p = 0.01).

Analyzing data in univariate logistic regression, VE was the only factor significantly associated with LMD (OR 8.89, CI 2.98–26.47, p < 0.001). The periventricular localization was included in the multivariate analysis since it seemed to be associated with LMD, although not significantly (OR 2.1, CI 0.82–5.42, p = 0.121). Both forward and backward multivariate logistic regression analyses gave the same results and, thus, the same OR. Only VE showed a strong significant association with LMD (OR 8.36, CI 2.78–25.11, p < 0.001), whereas periventricular localization did not (OR 1.48, CI 0.54–4.02, p 0.436). A univariate logistic regression included the IDH status and the MGMT promotor methylation. Detailed results of logistic regression analyses are reported in Tables 2 and 3.

**Table 2:** Odds ratio with 95% confidence interval and p values after univariate analysis for association with LMD

Variable	P value	Odds ratio	95% CI
Ventricular entry	** < 0.001**	**8.89**	**2.98–26.47**
Periventricular location	0.121	2.1	0.82–5.42
Age	0.522	0.99	0.96–1.02
Male sex	0.315	0.67	0.31–1.44
MGMT methylation	0.613	0.81	0.37–1.78
IDH wild-type	0.417	2	0.37–10.69
ATRX mutation	0.463	0.41	0.04–4.33

**Table 3:** Odds ratio with 95% confidence interval and p values after multivariate logistic regression

Variable	P value	Odds ratio	95% CI
Ventricular entry	** < 0.001**	**8.36**	**2.78–25.11**
Periventricular location	0.436	1.48	0.54–4.02

## Discussion

In this work, we aimed to clarify the influence of VE during GBM exeresis on the spread of glial tumor cells through CSF. LMD related to GBM is associated with shorter OS [5, 15, 22], thereby representing an adverse prognostic factor. We observed higher rates of LMD when VE was performed than in cases where this surgical event did not occur.

Our definition of LMD is based on a review of the literature and shared guidelines such as EANO and RANO [15, 33–36]. We decided to consider LMD when lesions disseminated along the subependymal zone, a leptomeningeal contrast enhancement around the gyri and sulci appeared, or multiple contours of nodular deposit in the subarachnoid space were detected: all of these occurrences have been observed to be associated with worse outcomes. We did not use the 5-ALA-derived fluorescence to detect the ventricular wall infiltration because its usage for ventricles is poorly understood and may not always represent tumor infiltration [37].

Indeed, a theoretical risk of false negatives concerning LMD is present since the CSF cytology or ctDNA in the blood sample would be the ultimate test to exclude actual disease spread [37]. However, such exams are not routinely performed, and MRI is the gold standard for GBM follow-up in clinical practice. Moreover, ctDNA blood sampling, despite its promising results so far, is still being investigated.

Cases of SVZ localization with subependymal spread were excluded. This subgroup could arguably be defined as subventricular since an apparent intraventricular spread is already present in these patients, and the ventricular violation by the tumor growth could intuitively bear higher rates of tumor cells spread through CSF.

The risks related to VE during GBM exeresis have been debated in the literature in the last few years [25, 36], but no univocal evidence has been reported, and few works dealt with this topic [20, 26, 38–40]. Jhon et al. [25] indicated that 50% of patients with VE during tumor resection had complications, with hydrocephalus being the most common [36, 41]. We decided not to investigate the onset of hydrocephalus. In fact, given the sequelae of the post-surgical treatments (i.e., brain atrophy, disease progression, side effects), it is hard to distinguish hydrocephalus from hydrocephalus ex vacuo and link potential neurological variations to the onset of hydrocephalus.

Moreover, our work aims to investigate the possible role of VE in causing progression in GBM patients. Typically hydrocephalus is quite common in these patients, but it does not strictly represent disease progression.

The metanalysis of Mistry et al. [16] collects all previous reports about VE and its potential consequences. The Authors found higher odds of developing LMD after VE [16] and also higher rates of complications in SVZ GBM [42] as the only independent variable associated with post-surgical LMD [26]. Young et al. [20] have shown that VE was not associated with worse outcomes and LMD. Because of the insufficient evidence in the literature, neurosurgeons based their surgical strategies on their own experience.

Our results confirm the hypothesis [18, 19] of glial tumor cell dissemination through CSF. We observed higher rates of LMD after VE, with similar results in the subgroup of periventricular GBM. We also confirmed that periventricular GBMs without VE have no higher rates of post-surgical LMD. These results seem to challenge recent findings [20, 26], probably due to higher rates of VE during surgical exeresis in our series (51%) compared to the literature (Young 26.5% [20] and Mistry 36.6% [26]). Different neurosurgeons in different Institutions and the consequent different decision-making strategies influenced the results. There is no standard protocol for performing VE in our Institution, but the neurosurgeon was the same in all cases.

The literature has reported that SVZ localization of GBM is a poor prognosis predictor [43–46] and is associated with a high rate of LMD. According to our data, GBMs not transgressing the ventricular wall (without apparent subependymal involvement) is not associated with a higher rate of post-surgical LMD (p > 0.1). This divergence is because there is an overlap in casting cases of VE and periventricular GBM. After all, the VE is achieved more frequently when GBM is periventricular to reach maximal EOR.

GBM genotype has also been considered in our analysis but based on our data, IDH or MGMT promoter status was not correlated to a higher risk of LMD.

Some features of our MRI analysis have to be discussed. Many of the patients in our study performed preoperative 1.5 T MRI and postoperative 3 T MRI. We have included these patients in our work because the tumor's morphological characteristics (volume, contrast enhancement) are similar between 1.5 and 3 T MRI [47]. Another point is the definition we adopted of LMD as a discontinuous abnormal FLAIR signal combined with a contrast-enhancing portion. Increased contrast enhancement detected by MRI just after or during treatment can be produced by several causes, such as postoperative changes, microischemic lesions, and treatment-associated inflammation [48]. Therefore, we considered combining FLAIR and T1-weighted contrast-enhanced MRI to have higher accuracy in identifying LMD.

T1-weighted contrast-enhanced MRI should be used within two days after surgery to assess the EOR and no later than 72 h after the operation. The immediate postoperative MRI in GBM is not routinely done in our Institution, and the early postoperative MRI is at least one-month post-surgery, thereby preventing an exact estimation of the EOR. We excluded patients with disease recurrence at early postoperative MR (i.e., a contrast-enhancing area or FLAIR signal alterations in the surgical cavity). Thus, since only patients with complete tumor removal at early postoperative MRI were considered, no relevant differences in EOR in VE and non-VE groups occurred, and both groups were homogeneous in this respect.

The correlation between VE and the EOR is a relevant aspect. In the literature, the role of gross total resection (GTR) over subtotal resection in progression-free and overall survival is accepted [9, 10], while the superiority of supra-total resection (SpTR) over GTR is less clear [11, 49–51] [57], but has given some promising results. In our series, GTR was the main goal in each case, and the VE has sometimes been performed in not-periventricular GBM, even if the tumor limits were not adjacent to the ventricular walls, to obtain a supratotal resection (SpTR). In the case of apparent subependymal involvement, the patient's prognosis is very scarce [52, 53]. The survival time would probably not be enough to evidence any LMD associated with the VE. In these cases, the maximal EOR could be a positive prognostic factor, and in the cases of SpTR, a possible VE should not be a limit for a wider EOR. However, there are borderline cases in which the VE should be avoided. For instance, in cases of periventricular localization of GBM, without an apparent subependymal involvement, neurosurgeons should be aware of the consequences of VE instead of the possible reach of the supra total resection. In our opinion, EOR must be the primary goal of GBM exeresis but avoiding VE when possible should be another relevant issue.

The principal limitations of our work are its retrospective, non-randomized nature and the insufficient number of patients' spine MRIs pre-or post-surgical exeresis of GBM: the latter point might have brought an underestimation of actual cases of LMD. However, spine imaging is not routinely done as a follow-up investigation in GBM patients unless spinal symptoms develop. Therefore, the topic of spinal LMD in GBM has received only limited attention in the neurosurgical and neuro-oncological debate.

The influence of all the variables investigated in this work on the patients' OS was not investigated. Many other factors, such as adjuvant therapy, molecular patterns, and the size of GBM, can influence survival rates. Despite this, the role of LMD as an adverse prognostic factor on OS is accepted [5, 15, 22].

## Conclusion

According to our data, VE during surgical exeresis of GBM increases the rate of post-surgical LMD. Thus, neurosurgeons should avoid VE when feasible to prevent this disease progression, potentially influencing OS negatively. This statement does not override the need for maximal EOR, which must remain the goal of GBM surgery because as one of the foremost positive prognostic factors. Further studies should be oriented to the specific risk of LMD associated with GTR and SpTR groups of patients.
